# Opioid Overdose With Parkinsonian Features

**DOI:** 10.5811/cpcem.2019.7.43537

**Published:** 2019-10-14

**Authors:** Paul Cohen, Jason B. Hack

**Affiliations:** * Brown University, Department of Emergency Medicine, Providence, Rhode Island; † Brown University, Department of Emergency Medicine, Division of Medical Toxicology, Providence, Rhode Island

## Abstract

A 54-year-old man presented to the emergency department with confusion and Parkinsonian features after suspected heroin snorting. He had magnetic resonance imaging of the brain demonstrating isolated symmetric bilateral globus pallidus (GP) restricted diffusion and edema consistent with hypoxic ischemic encephalopathy. In contrast to other anoxic/ischemic insults, where the GP is preferentially spared, autopsy reports on intravenous heroin users have found the GP to be specifically affected, often demonstrating symmetric bilateral lesions. Opioid toxicity should be considered in patients presenting with Parkinsonian features on examination or pallidal lesions on imaging, especially in younger adults where infarction is less common.

## CASE PRESENTATION

**Image 1:**
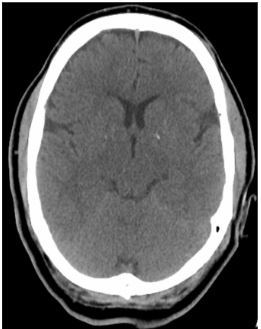
Computed tomography scan of the brain without contrast obtained hours earlier without evidence of globus pallidus ischemia.

**Image 2:**
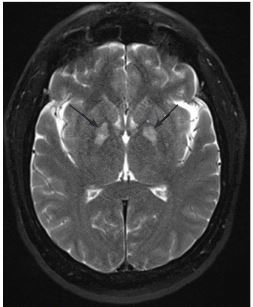
Brain magnetic resonance T2/fluid attenuated inversion recovery with evidence of bilateral globus pallidus ischemia (arrows).

A 54-year-old man presented to the emergency department (ED) after receiving naloxone for a suspected opioid overdose. He was found in bed unresponsive, hypoxemic to SpO_2_ 83%, with sonorous respirations and presumed heroin powder on his face and bedside table. On arrival to the ED he was awake but confused and noted to have bilateral upper and lower extremity rigidity along with sustained clonus, hyperreflexia in upper and lower extremities, and complained of “foot cramping.” A non-contrast computed tomography of the brain was normal (Image 1). Brain magnetic resonance imaging obtained 15 hours later demonstrated isolated symmetric bilateral globus pallidus (GP) restricted diffusion and edema (Image 2).

## DIAGNOSIS

In autopsies on patients with heroin use disorder, bilateral GP lesions were noted to be frequently encountered.^1^ This finding is atypical compared to other anoxic-ischemic insults, where the GP is preferentially spared as opposed to the caudate.^2^ This GP insult was once thought to be unique to carbon monoxide poisoning, but its causal etiology has expanded to include such conditions as methanol poisoning, cyanide poisoning and, increasingly recognized, opioid overdose. The mechanism behind this finding is not understood, although it has been speculated to be secondary to recurrent episodes of cerebral hypoxia as opposed to a direct neurotoxic substance. Lesions to the GP typically cause akinetic-rigid and/or dystonic syndromes such as noted in this patient.

The patient was admitted to the hospital where he admitted to snorting heroin powder. He had resolution of his symptoms by hospital day five. Hypoxic ischemic insult, including opioid toxicity, should be considered in patients with Parkinsonian features on exam and/or isolated pallidal lesions on imaging without history of overt cardiorespiratory arrest, especially in adolescents and younger adults where infarction is less common.

**CPC-EM Capsule**

What do we already know about this clinical entity?

*Bilateral Globus Pallidus (GP) lesions have been noted in autopsies of patients with heroin use disorder. The etiology is unclear as anoxic-ischemic insults typically affects other brain areas*.

What is the major impact of the image(s)?

*Toxin induced Globus pallidus injury is a recognized finding in carbon monoxide, methanol, and cyanide poisoning. This magnetic resonance imaging finding should prompt a differential that includes opioid use*.

How might this improve emergency medicine practice?

*Recognition of GP lesions may identify a patient at high risk of future opioid related injury or death; who would benefit from counseling, substance use referral, and other resources*.
